# The immune checkpoint TIM-3/HMGB-1 axis in myocardial infarction

**DOI:** 10.1038/s44325-025-00061-x

**Published:** 2025-06-27

**Authors:** Laura I. Yousif, Aukje G. Sijtema, Ymke Appels, Elles M. Screever, Irene V. van Blokland, Roy Oelen, Hilde E. Groot, Tamás G. Gergely, Márton Kocsis, Przemyslaw Leszek, Zoltán V. Varga, Joseph Pierre Aboumsallem, Pim van der Harst, Lude H. Franke, Monique G. P. van der Wijst, Rudolf A. de Boer, Erik Lipšic, Wouter C. Meijers

**Affiliations:** 1 Erasmus MC, Cardiovascular Institute, Thorax Center, Department of Cardiology, Rotterdam, The Netherlands; 2 University Medical Center Groningen, Department of Cardiology, Groningen, The Netherlands; 3 University Medical Center Groningen, Department of Genetics, Groningen, The Netherlands; 4 Center for Pharmacology and Drug Research & Development, Department of Pharmacology and Pharmacotherapy, Semmelweis University, Budapest, Hungary; 5 MTA-SE Momentum Cardio-Oncology and Cardioimmunology Research Group, Budapest, Hungary; 6 Department of Heart Failure and Transplantology, Cardinal Stefan Wyszyński National Institute of Cardiology, Warszawa, Poland; 7 University Medical Center Utrecht, Department of Cardiology, Utrecht, The Netherlands

**Keywords:** Heart failure, Acute inflammation

## Abstract

Immune checkpoints are understudied in cardiovascular disease. We investigated the TIM-3 pathway in human serum, peripheral blood mononuclear cells (PBMCs) and cardiac tissue following myocardial infarction (MI). First, TIM-3 ligands in serum, galectin-9 and HMGB-1, were associated with cardiac remodelling 4 months post-MI (*n* = 357). Next, post-hoc single-cell RNA-sequencing of PBMCs from MI patients (*n* = 38) and controls (*n* = 38) revealed acute downregulation of TIM-3 in lymphocytes 24 h post-MI, which occurred after 8 weeks in myeloid cells. In the heart, single-nucleus RNA-sequencing and spatial transcriptomics of MI tissue demonstrated cardiomyocyte HMGB-1 upregulation which could communicate with myeloid TIM-3. Pro-inflammatory macrophages specifically showed significant TIM-3 expression and NLRP3 inflammasome activity. On the protein level, HMGB-1 was also upregulated in the infarcted heart and actively translocated throughout the cells. Finally, in vitro macrophage stimulation with HMGB-1 induced pro-inflammatory polarization (e.g. NLRP3 pathway activation), which was prevented by blocking TIM-3. Thus, TIM-3/HMGB-1 interaction presents as a target in cardiac inflammation following MI.

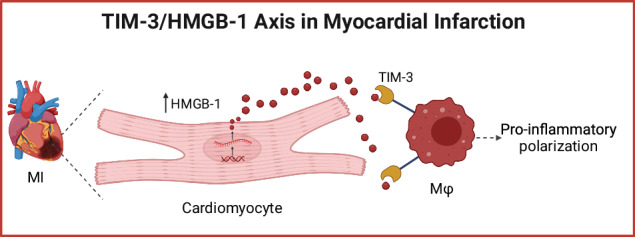

## Introduction

Immune checkpoints (ICs) serve as switches on immune cells to induce inhibition and regulate the immune response, and modulation with IC inhibitors has been a breakthrough in cancer treatment^1–3^. While the immune system is essential in the response following a myocardial infarction (MI), the role and regulatory mechanisms of ICs in this context remain unknown.

The two most studied ICs are cytotoxic T-lymphocyte associated protein-4 (CTLA-4) and programmed cell death protein-1 (PD-1), owing to their widespread and effective use as IC inhibitors in oncology^4–6^. However, a novel IC gaining attention is T-cell immunoglobulin and mucin-domain containing-3 (TIM-3). TIM-3 is functionally and biologically more complex than CTLA-4 and PD-1, being expressed by a variety of both innate and adaptive immune cells and having multiple binding sites for its many ligands^7,8^. Until now it has been suggested that it might be pro-inflammatory in the resting, naive immune system but anti-inflammatory in the active, adaptive immune system.

Noteworthy is that some TIM-3 ligands, such as high mobility group box-1 (HMGB-1), are associated with cell damage or death^9^. HMGB-1 is a non-histone chromatin-associated protein, and a known danger associated molecular pattern, or DAMP. It is released from the cell upon cellular damage or death, and acts as an endogenous signal to promote and exacerbate inflammation. Other recognized TIM-3 ligands are apoptosis marker phosphatidylserine (PtdSer), carcinoembryonic antigen-related cell adhesion molecule 1 (CEACAM1), and galectin-9 (Gal-9)^9^. Our group has previously demonstrated that in serum of patients with worsening heart failure, TIM-3 ligands are significantly associated with severity, and predicted hospitalization and all-cause mortality^10^. In this study we assessed CTLA-4, PD-1 and TIM-3 ligands association with cardiac remodeling, and studied the involvement of TIM-3 in the infarcted myocardium.

## Results

### Circulating levels of TIM-3 ligands are associated with cardiac remodeling after MI

Baseline characteristics of the Glycometabolic Intervention as adjunct to Primary percutaneous intervention in ST elevation myocardial infarction (GIPS)-III trial patients have previously been reported^11^. In summary, the mean age was 59 ( ± 12) years and 25% of the patients were female. The median ischemia time was 159 (interquartile range [IQR] 108, 242) minutes and treatment with metformin did not impact left ventricular ejection fraction (LVEF) at 4 months. To determine association of circulating TIM-3 ligands with cardiac remodeling post-MI, levels of HMGB-1, Gal-9 and CEACAM1 were measured in serum of 357 patients available for these analyses. All three ligands demonstrated significant associations with cardiac remodeling. In univariate regression analyses, serum levels of Gal-9 at 24 h post-MI (5.69 ± 1.80 ng/mL) were associated with an increased infarct size (*β* = −0.258, *p* = 0.006), and serum levels of HMGB-1 (24.56 ± 8.53 ng/mL) and CEACAM1 (19.69 ± 10.28 ng/mL) were associated with lower LVEF (*β* = −0.210, *p* = 0.026 and *β* = −0.189, *p* = 0.040, respectively) 4 months post-MI (Table 1). In multivariate linear regression analyses, correcting for age, sex, body mass index (BMI), hypercholesterolemia, Thrombolysis in Myocardial Infarction (TIMI) flow and myocardial blush grade, Gal-9 and HMGB-1 remained significantly associated with cardiac remodeling parameters (*β* = −0.254, *p* = 0.015 and *β* = −0.236, *p* = 0.018, respectively).

**Table 1:** Associations of circulating TIM-3 ligands with cardiac remodeling

	*Infarct size*				*LVEF*			
	Univariate		Multivariate		Univariate		Multivariate	
	*β*	*p-value*	*β*	*p-value*	*β*	*p-value*	*β*	*p-value*
***HMGB-1***	-0.006	0.947	-	-	-0.210	0.026	-0.236	0.018
***Gal-9***	-0.258	0.006	-0.254	0.015	0.057	0.535	-	-
***CEACAM1***	0.036	0.696	-	-	-0.189	0.040	-0.105	0.251

Linear and multivariate regression of HMGB-1, Gal-9, CEACAM1 at 24 h post-MI (quartile(Q) 4 vs Q1). Multivariate regression adjusted for age, sex, body mass index (BMI), hypercholesterolemia, Thrombolysis in Myocardial Infarction (TIMI) flow and myocardial blush grade. LVEF Left Ventricular Ejection Fraction; TIM-3 T-cell Immunoglobulin and Mucin-Domain Containing Protein-3; HMGB-1 High-Mobility group box 1; Gal-9 Galectin-9; CEACAM1 Carcinoembryonic-Antigen-related Cell-Adhesion Molecule 1.

In an exploratory approach, we also assessed the associations of cardiac remodeling parameters with established ligands of PD-1 (PD-L1 and PD-L2) and CTLA-4 (CD80 and CD86), which did not yield significant results (Supplementary table 1).

### In peripheral blood, lymphoid cells downregulate TIM-3 expression in the acute phase post-MI while myeloid cells maintain it

While circulating IC ligands might be indicative of outcome and could potentially hold biomarker potential, it is also of interest to examine IC receptor expression on circulating immune cells and whether this is affected by an MI. Given that TIM-3 ligands in the serum were the only ones among the three most pharmacologically relevant ICs to be associated with cardiac remodeling after MI, and a select few are known to be markers of cell death and injury (e.g. HMGB-1 and PtdSer), we decided to further investigate this pathway. Therefore, we performed post-hoc analysis on single-cell RNA sequencing (scRNAseq) data to assess expression of TIM-3 gene levels, encoded by *HAVCR2*, in PBMCs from patients with an MI compared to a control cohort. Baseline characteristics of these patients are presented in the original publications^12,13^. In short, the mean age of the two groups was relatively similar, 59 ± 12 years for the controls and 60 ± 11 years for the MI patients, and both comprised of 6 (16%) female patients. In the MI group, 3 (8%) patients had hypertension, and the median ischemia time was 127.5 (IQR 96, 151) minutes.

**Fig. 1:**
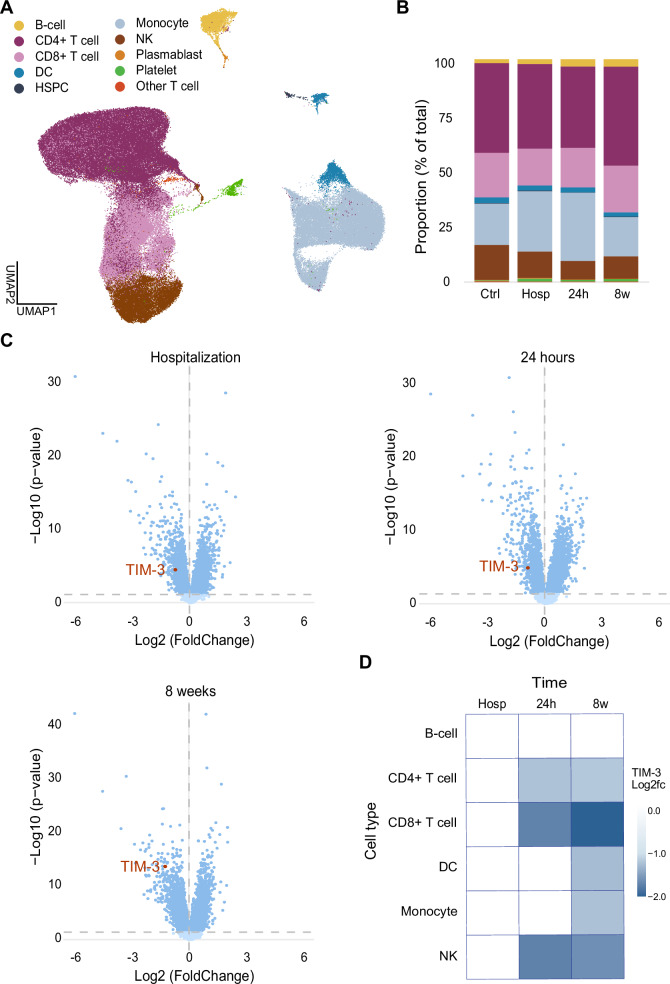
scRNAseq of PBMCs post-MI. **A** Identification of 10 cell populations. **B** Cell population proportions over time. **C** Differential gene expression of PBMCs showing that TIM-3, encoded by HAVCR2, was downregulated in PBMCs of patients with MI up to 8 weeks post-MI as compared to in PBMCs from the healthy controls (*p*-value < 0.05). **D** A temporal overview indicating that effector cells, CD4+ and CD8+ T cells as well as NK cells drove this decreased phenotype at 24 hours post-MI, and that all cells, except B-cells, downregulated TIM-3 gene expression after 8 weeks in patients with MI as compared to controls. MI Myocardial infarction; TIM-3 T-cell immunoglobulin and mucin-domain containing protein-3; PBMCs Peripheral blood mononuclear cells; NK Natural killer cells; DC Dendritic cells.

scRNAseq revealed 10 major cell populations (Fig. 1A and Supplementary Fig. 1). T cells were the most abundant cell types, but monocytes and natural killer (NK) cells seemed to increase and decrease in numbers the most, respectively, in MI patients at 24 hours post-MI as compared to controls (Fig. 1B and Supplementary Data Table 1). To determine whether TIM-3 expression was significantly altered in the peripheral blood of MI patients, we performed a differential gene expression (DGE) analysis on the 10 cell populations and found that it was significantly downregulated across all three time points (Fig. 1C and Supplementary Data Table 2).

To identify which immune cells drove the expression pattern, we performed DGE on the individual cell types over time. Our initial finding was that TIM-3 was differentially expressed in 6 out of 10 populations; B-cells, CD4^+^ and CD8^+^ T cells, dendritic cells (DCs), monocytes and NK cells, and that the downregulation at hospitalization was not driven by just one cell type (Fig. 1D and Supplementary Data Table 3). Furthermore, we observed that lymphoid cells (T cells and NK cells) had decreased TIM-3 expression at 24 hours, while myeloid cells (DCs and monocytes) maintained their TIM-3 expression until 8 weeks, at which it became significantly downregulated.

### TIM-3 and its ligands expressed at different regions during myocardial infarction

**Fig. 2:**
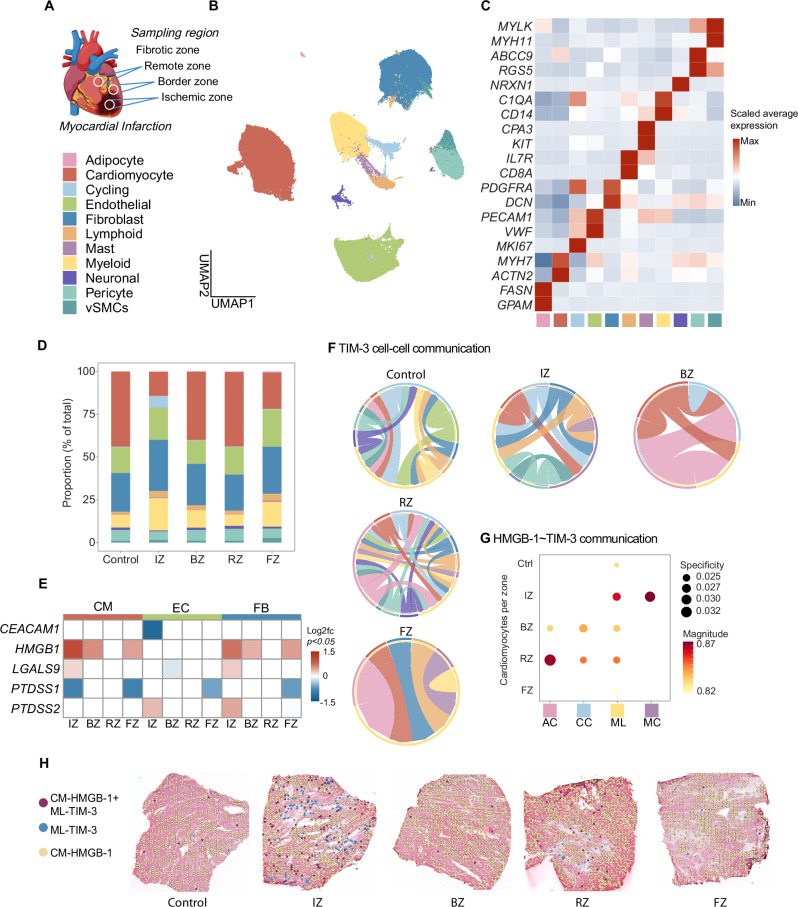
snRNAseq of human cardiac tissue from different zones of an MI. **A–C** Identification and validation of cell populations from each sampling region. **D** Overview of total cell proportions in control versus different zones. **E** Differential gene expression of TIM-3 ligands by cardiomyocytes (CM), endothelial cells (EC) and fibroblasts (FB). **F** Ligand-receptor Analysis frAmework, LIANA, chord diagrams depicting which cell types were predicted to receive signals from TIM-3. **G** Cardiomyocytes specific communication through HMGB-1, encoded by *HMGB1*, with TIM-3 on adipocytes (AC), cycling cells (CC), myeloid cells (ML) and mast cells (ML). **H** Spatial transcriptomics showing colocalization of CM expressing HMGB-1 with myeloid cells expressing TIM-3. snRNAseq Single-nucleus RNA sequencing; MI Myocardial infarction; HMGB-1 High mobility group box-1; Ctrl Control; IZ Ischemic zone; BZ Border zone; RZ Remote zone; FZ Fibrotic zone; HMGB-1 High mobility group box-1; TIM-3 T-cell immunoglobulin and mucin-domain containing protein-3.

Next, we investigated the expression of TIM-3 and its ligands in human myocardial tissue from patients with an MI. Single-nucleus RNA sequencing (snRNAseq) data from 31 samples (23 patients) from different regions of the heart (ischemic zone, border zone, remote zone, and fibrotic zone) as well as control tissue was publicly available (Fig. 2A)^14^. MI samples included cardiac tissue obtained at different time points after the onset of clinical symptoms (chest pain), before the patients received an artificial heart or a left-ventricular assist device due to cardiogenic shock, or as a bridge until transplantation^14^. Ischemic zone, border and remote samples were obtained within 15 days of the MI. Fibrotic zone tissue included samples from later stages (30–200 days), exhibiting ischaemic heart disease, from heart transplantation recipients at the time of orthotopic heart transplantation. Upon quality control and integration of data, 11 major cell populations were identified as shown in figure (Fig. 2B, C). The lowest number of cardiomyocytes was observed in the ischemic and fibrotic zones, likely due to cell death (Fig. 2D and Supplementary Data Table 4). Myeloid and lymphoid cell populations were increased in the ischemic zone, and a specific population of cycling cells, enriched for proliferation marker *MKI67*, was abundant in the ischemic zone, suggesting marked proliferation of various cell types in the ischemic zone as opposed to solely cell death.

DGE analysis comparing the different sampling regions to control tissue revealed RNA expression of TIM-3 ligands by cardiomyocytes, endothelial cells and fibroblasts (Fig. 2E).

Cardiomyocytes showed upregulation of HMGB-1, encoded by *HMGB1*, in all zones except the remote zone, with highest expression in the ischemic zone. Gal-9, encoded by *LGALS9*, was only upregulated in the ischemic zone, and phosphatidylserine synthase 1 (PSS1), encoded by *PTDSS1*, which catalyses phosphatidylcholine into phosphatidylserine, was downregulated in both the ischemic and fibrotic zone. Fibroblasts revealed similar results, with the exception of no significant PSS1 reduction in the infarct zone and an increased expression of phosphatidylserine synthase 2 (PSS2), encoded by *PTDSS2*, which catalyses phosphatidylethanolamine into phosphatidylserine, in the ischemic zone. Endothelial cells, on the other hand, did not show any significant alteration in HMGB-1 expression. Instead, CEACAM1 expression, encoded by *CEACAM1*, was significantly decreased in the infarct zone and Gal-9 in the border zone. Furthermore, as seen in fibroblasts, endothelial cell expression of PSS1 was decreased in the fibrotic zone and PSS2 expression increased in the ischemic zone. Regarding TIM-3 expression among the immune cells, only myeloid cells showed a significant decrease in TIM-3 expression in the fibrotic zone (Supplementary Fig. 2).

### TIM-3/HMGB-1 interaction is prominent between myeloid cells and cardiomyocytes

To explore intracellular crosstalk between TIM-3 and its ligands, a ligand-receptor analysis called LIANA was performed. While cardiomyocytes, endothelial cells and fibroblasts all differentially expressed TIM-3 ligands, inferred ligand-receptor interaction with TIM-3 was only consistent with cardiomyocytes throughout all zones (Fig. 2F and Supplementary Data Table 5). This interaction occurred most prominently and constitutively with TIM-3 expressed on myeloid cells. Besides myeloid cells, there was also a predicted interaction between cardiomyocytes and TIM-3 on mast cells in the ischemic zone, and with adipocytes and cycling cells in the border zone. Next, we assessed inferred predictions on ligand-receptor level which revealed that cardiomyocytes were predicted to solely communicate with TIM-3 via HMGB-1. Also, it was evident that the interaction with myeloid cells occurred throughout the heart during an MI, with the most signaling between the two occuring in the ischemic zone (Fig. 2G). TIM-3 on adipocytes and cycling cells could interact with HMGB-1 from cardiomyocytes in the border and fibrotic zone, whereas mast cells only seemed to mediate this interaction in the ischemic zone. Furthermore, as a DAMP, HMGB-1 is known for its signalling through toll-like receptor 4 (TLR4) or (Receptor for Advanced Glycation End-products) RAGE in the heart. However, ligand-receptor analysis did not yield significant predicted interactions between HMGB-1 and RAGE in any of the zones. Significant TLR4 interactions were inferred between cardiomyocyte-HMGB-1 and TLR4 on myeloid cells in the remote and fibrotic zone, but not in the ischemic or border zone. This suggests a specific role for cardiomyocyte-HMGB-1 and myeloid-TIM-3 interaction in the ischemic and border zones – the site where most injury and inflammation occurs.

Finally, we were interested in the colocalization of the predicted ligand-receptor interactions, and so the next step was to determine spatial colocalization of cardiomyocytes expressing HMGB-1 and myeloid cells expressing TIM-3. An identical pattern between cardiomyocyte-HMGB-1 and myeloid-TIM-3 interaction was observed as seen in the ligand-receptor interactions (Fig. 2H). Namely, that there was colocalization of the two in all regions of the infarcted heart as well as in control tissue, but most predominantly in the ischemic zone.

These findings suggest a role for cardiomyocytes/HMGB-1 binding to myeloid/TIM-3 in the ischemic and border zone of the infarcted heart.

### Increased TIM-3 expression in activated, pro-inflammatory macrophages in the ischemic zone

**Fig. 3:**
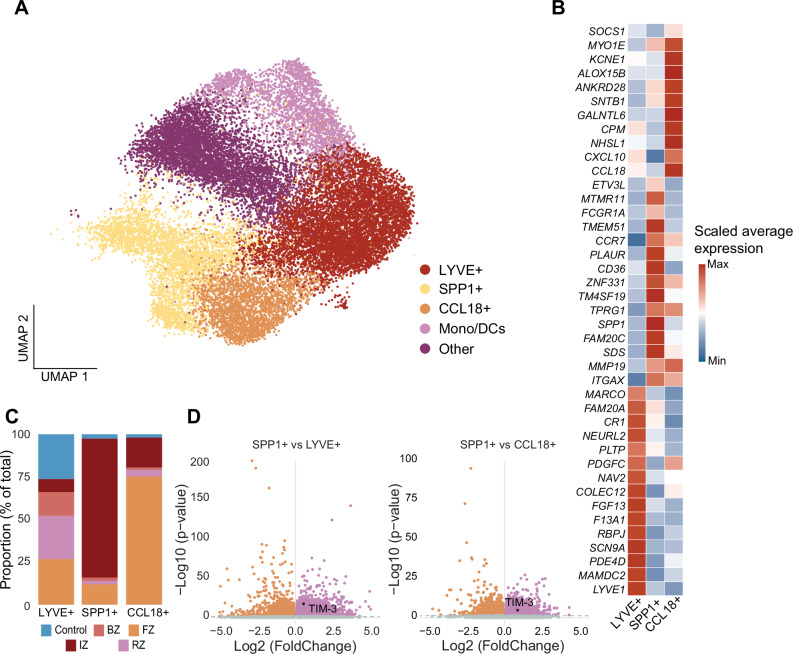
Macrophage TIM-3 expression in the ischemic zone. **A** UMAP embedding of snRNAseq data colored by clusters. **B** Heatmap with marker genes for macrophage clusters based on *LYVE*, *SPP1* and *CCL18* expression. **C** Total cell proportions (in percentage) of *LYVE* + *, SPP1+* and *CCL18+* macrophages across the different zones of the infarcted heart and in healthy controls. **D** Differential gene expression of TIM-3 comparing *SPP1+* macrophages with *LYVE+* and *CCL18+* macrophages in the IZ. snRNAseq Single-nucleus RNA sequencing; IZ Ischemic zone; BZ Border zone; RZ Remote zone; FZ Fibrotic zone; TIM-3 T-cell immunoglobulin and mucin-domain containing protein-3.

Given the prominent interaction between cardiomyocytes/HMGB-1 and myeloid/TIM-3, as well as the hypothesis in literature that TIM-3 can modulate macrophage polarization, we explored TIM-3 expression by macrophages in the ischemic zone from the snRNAseq. Five distinct cell clusters were identified within the myeloid cells: three macrophage clusters, one monocyte and classical DC cluster, and another cluster with more ambiguous myeloid gene expressions (other) (Fig. 3A, B, Supplementary Fig. 3 and Supplementary Data Table 6). Macrophage clusters were further divided among resident (*LYVE* + ), *SSP1*+ and *CCL18*+ macrophages. *SPP1*, encoding for the protein osteopontin, has been related to activated and pro-inflammatory macrophages important for post-MI remodeling^15^. As this cluster also showed increased *CD36* and *PLAUR* expression, it illustrates highly active, phagocytic and pro-inflammatory cells involved in tissue invasion and recruitment^16^. *CCL18* encodes for chemokine CCL18 which is known for recruiting regulatory T-cells to dampen the inflammation, thus these macrophages are more associated with a reparative, anti-inflammatory/M2 profile^14,15,17,18^. Resident macrophages were present in all zones of the infarcted heart, whereas *SSP1+* macrophages were predominantly present in the ischemic zone and the *CCL18+* macrophages in the fibrotic zone, suggesting that the first are more involved in the acute phase and the latter in the reparative phase following an MI (Fig. 3C). Gene-Ontology (GO) pathway analysis revealed that *positive regulation of NLRP3 inflammasome complex assembly* (adjusted *p* value = 0.0173, [FDR] = 0.0132) and *positive regulation of inflammasome-mediated signaling pathway* (adjusted *p* value = 0.0339, FDR = 0.0258) were significantly upregulated in *SPP1+* compared to resident macrophages, demonstrating inflammatory responses and a pathway of interest (Supplementary Data Table 7). Additionally, comparison between the three macrophage clusters revealed that TIM-3 was significantly more expressed in *SSP1+* macrophages compared to both *LYVE+* and *CCL18+* macrophages (Fig. 3D and Supplementary Data Table 8).

### HMGB-1 stimulation of macrophages in vitro induces M1 polarization

**Fig. 4:**
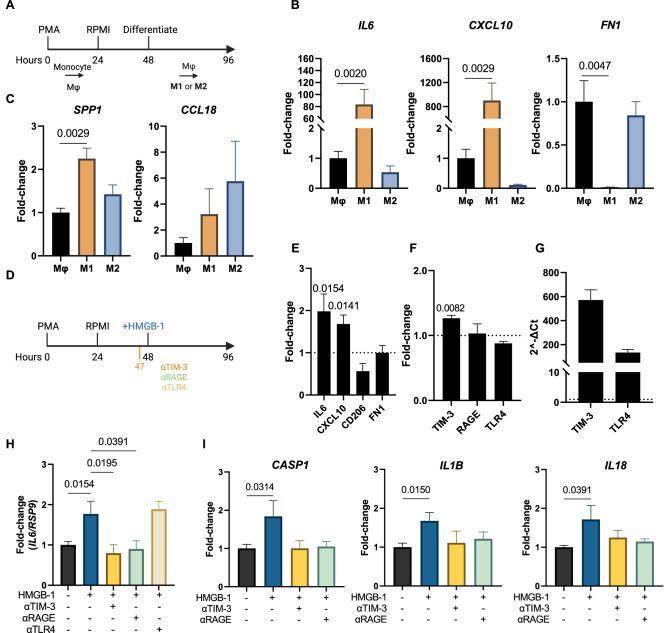
In vitro characterization of HMGB-1 stimulated macrophages. **A** In vitro model of macrophage differentiation from monocytes to unstimulated macrophage (Mφ) and then to pro-inflammatory (M1) or anti-inflammatory (M2) macrophages. **B** Gene expression of classical M1 (*IL6* and *CXCL10*) and M2 (*FN1*) markers. **C** Expression of *SPP1* and *CCL18* showing M1 and M2 mimicking of transcriptomics macrophage clusters. **D** Schematic of HMGB-1 and receptor blockade experiments. **E** Expression of M1 and M2 macrophage markers in macrophages stimulated with 5 µg/mL HMGB-1 for 48 hours. **F** TIM-3, RAGE and TLR4 expression in macrophages stimulated with 5 µg/mL HMGB-1 for 48 hours. **G** Quantification of TIM-3 and TLR-4 expression in macrophages stimulated with HMGB-1 compared to RAGE based on the 2^-ΔCt^. **H** Expression of *IL6* upon stimulation with 5 µg/mL HMGB-1 for 48 hours with or without preincubation of 10 µg/mL neutralizing antibodies against TIM-3, RAGE or TLR4. **I** Expression of inflammasome pathway markers *CASP1, IL1B* and *IL18*. qPCR expression was normalized by *RSP9* and data is presented as mean ± standard error of the mean (SEM). HMGB-1 High mobility group box-1; TIM-3 T-cell immunoglobulin and mucin-domain containing protein-3; RAGE Receptor for advanced glycation endproducts; TLR4 toll-like receptor 4.

Evidence supporting a pro-inflammatory interaction between HMGB-1 from cardiomyocytes in the most affected zones of the infarcted heart with TIM-3 on macrophages warranted in vitro validation. Therefore, we set out to determine the effect of HMGB-1 stimulation on macrophages in vitro. First, we differentiated macrophages from THP1 monocytes with 150 nM Phorbol 12-myristate 13-acetate (PMA) for 24 hours as illustrated in Fig. 4A. This was followed by fresh medium for 24 hours to obtain unstimulated macrophages (Mφ). Next, we recapitulated pro-inflammatory (M1) and anti-inflammatory (M2)-like macrophage culture through stimulation with 10 ng/mL Lipopolysaccharide (LPS) and 20 ng/mL interferon γ (IFNγ) or 20 ng/mL interleukin (IL)-4 and 20 ng/mL IL-13, respectively, for 48 hours. Macrophage polarization was assessed by measuring classical M1 (*IL6* and *CXCL10*) and M2 (*FN1*) gene markers. LPS and IFNγ stimulation significantly increased expression of *IL6* and *CXCL10*, but not *FN1*, confirming a pro-inflammatory M1-like profile (Fig. 4B). Similarly, cells stimulated with IL-4 and IL-13 revealed low levels of *IL6* and CXCL10 but a relative maintained expression of *FN1*, confirming anti-inflammatory polarization. Interestingly and in line with the transcriptomics data, M1 macrophages showed significant upregulation of *SSP1*, whereas *CCL18*, was substantially more expressed by M2 macrophages (Fig. 4C). To investigate whether HMGB-1 could induce M1 polarization, Mφ were stimulated with 0 or 5 µg/mL HMGB-1 for 48 hours (Fig. 4D). HMGB-1 stimulation induced M1-like polarization of macrophages in vitro as presented by significantly upregulated *IL6* and *CXCL10*, a slight decrease in *CD206* and without any significant alteration in *FN1* (Fig. 4E). TIM-3 gene expression, not RAGE or TLR4, was slightly significantly upregulated upon HMGB-1 stimulation (Fig. 4F). Furthermore, upon comparing expression of the three receptors in macrophages stimulated with HMGB-1, TIM-3 and TLR4 were substantially more expressed than RAGE (Fig. 4G).

### Macrophage polarization by HMGB-1 is receptor-specific

HMGB-1 is known to drive pro-inflammatory responses in cells through interactions with its most described receptors, RAGE and TLR4. To demonstrate that HMGB-1-induced M1-polarization of macrophages can also result from binding of TIM-3, prior to stimulation with HMGB-1, Mφ were incubated with neutralizing antibodies for TIM-3, TLR4 or RAGE. The increase in *IL-6* was completely prevented by blocking TIM-3 (*p* = 0.0154) or RAGE (*p* = 0.0391), but not TLR4, suggesting that in this setting HMGB-1-induced M1-polarization occurs through TIM-3 or RAGE signalling (Fig. 4H). HMGB-1 as a DAMP is known to activate the NLRP3 pathway and result in the cleavage of caspase-1 and subsequent secretion of pro-inflammatory cytokines IL-1β and IL-18. To evaluate whether downstream signalling of TIM-3/HMGB-1 interaction in Mφ in comparison to RAGE was mediated though the NLRP3 pathway, gene expression of NLRP3 products was assessed. HMGB-1 stimulation increased expression of *CASP1* (*p* = 0.0424), *IL1B* (*p* = 0.0213) and *IL18* (*p* = 0.0319), all of which was prevented by pre-emptively blocking interaction with TIM-3 as well as RAGE (Fig. 4I). These findings demonstrate the pro-inflammatory response following TIM-3/HMGB-1 binding, possibly through NLRP3 signalling, and present this interaction as a target in cardiac inflammation following an MI.

### Increased HMGB-1 protein expression and translocation in murine and human infarcted myocardium

**Fig. 5:**
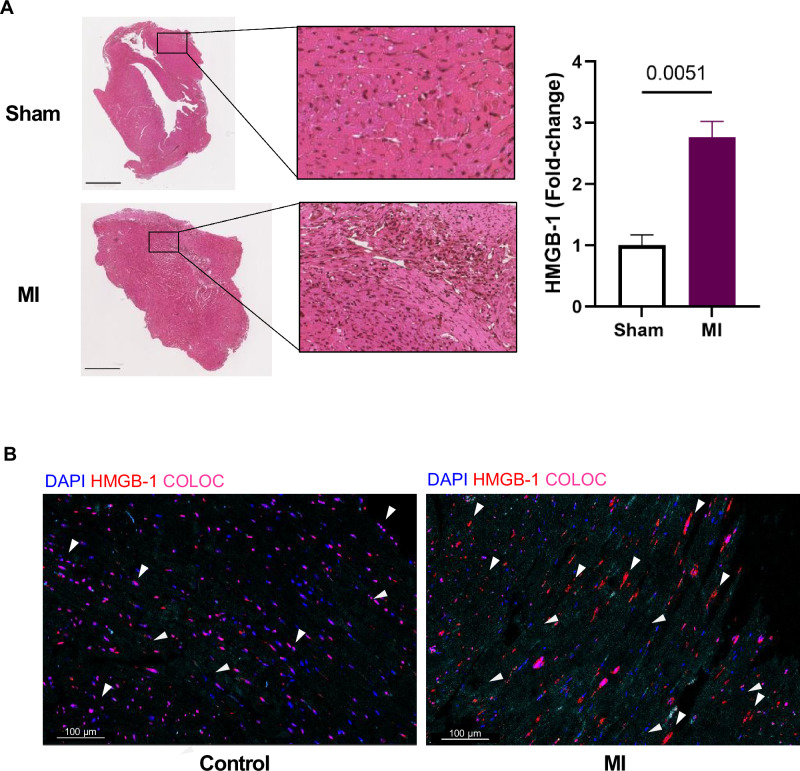
Myocardial HMGB-1 expression in ischemic injury. **A** Representative immunohistochemical staining of HMGB-1 on cardiac tissue from mice who underwent sham (*n* = 3) or MI (*n* = 3) surgery 6 weeks post-surgery and quantification. Scale is 2 mm. **B** Representative immunohistochemical staining of HMGB-1 in hearts of patients with ischemic injury compared to controls. Samples collected from explanted heart. White arrows indicate HMGB-1 expression; intranuclear in the left panel and translocated into the cytoplasm or extracellular space in the right. Scale is 100 µm. Data are mean ± SEM. COLOC Colocalization; HMGB-1 High mobility group box-1; MI Myocardial infarction.

Finally, we wanted to determine whether HMGB-1 was also increased on protein level in the myocardium after an MI. Therefore, we measured HMGB-1 in the hearts of mice with an MI, induced through permanent ligation of the left anterior descending (LAD) artery. The model was previously characterized, showing decreased LVEF in the MI group as compared to the sham group, along with clear fibrotic scare tissue and an upregulation in genes associated with inflammation and fibrosis^19^. Immunohistochemistry (IHC) revealed significantly increased HMGB-1 protein expression (*p* = 0.0051) in the infarcted myocardium as demonstrated in Fig. 5A. We also observed that HMGB-1 appeared to be more distributed throughout the tissue as opposed to mainly being intranuclear. This was even more prominent in human tissue, where the same translocation of HMGB-1 from the nucleus to the cytoplasm and extracellular space in hearts of patients with ischemic injury compared to controls was seen (Fig. 5B). This is illustrated by the pink signal, signifying colocalization (COLOC) of nuclear staining (blue) and HMGB-1 (red) in the control hearts compared to all the separate HMGB-1 observed in the ischemic injury hearts. Together, this indicates that HMGB-1 is indeed upregulated at protein level in the infarcted heart and is actively being translocated throughout the cells, even a certain period post-MI, implicating a functional role.

## Discussion

We investigated, for the first time, expression of TIM-3 and its ligands in the inflammatory and reparative phases following MI in serum, PBMCs and cardiac tissue, and demonstrated that TIM-3 signalling plays a role after an MI. At the serum protein level, we demonstrated that TIM-3 ligands were associated with cardiac remodeling after an MI. On PBMC level, overall TIM-3 RNA expression was significantly downregulated. Interestingly, myeloid cells maintained TIM-3 expression throughout the acute phase. At the cardiac tissue level, we identified upregulation of HMGB-1 by cardiomyocytes, which was strongly predicted to interact with myeloid TIM-3 in the ischemic zone. Furthermore, we observed that pro-inflammatory macrophages significantly upregulated TIM-3 in this zone, exhibiting NLRP3 inflammasome activity, and that in vitro stimulation of macrophages with HMGB-1 drove M1 polarization with increased expression of NLRP3 products, which was prevented by TIM-3 blockade.

While PD-1 and CTLA-4 are by far the most studied ICs, we focused on TIM-3 as its ligands in serum of patients were significantly associated with parameters of cardiac remodeling and function after an MI. This may be explained by the characteristic function of each IC. Functionally, CTLA-4 has been linked to autoimmune inflammation, and its ligands, CD80 and CD86, appropriately are customarily found in lymph nodes and spleens, not in the circulation^6^. PD-1 ligands are more widely expressed throughout the body, both on circulating cells and in peripheral tissue, aiding in the inhibition of T-cell responses. PD-L1 tends to be more organ-bound, possibly to maintain tolerance upon immune cell infiltration, whereas PD-L2 can be expressed by various cells depending on the local microenvironment. Within cardiac disease, we have previously shown that circulating levels of PD-L1 and PD-L2 are associated with HF severity and can predict outcome. The latter was also true for Gal-9^10^. Likewise, a study found that Gal-9 was associated with both coronary artery disease as well as the severity of coronary artery stenosis, implicating it in both chronic and more ‘’ongoing” cardiac damage^20^.

On the other hand, a recent study found that elevated serum HMGB-1 levels were associated with MI with or without HF, but not with HF alone^21^. The authors also described a correlation of HMGB-1 with cardiac damage and inflammation biomarkers. In another study, HMGB-1 was associated with non-calcified plaque burden in patients with coronary artery disease^22^. Given that HMGB-1 is a DAMP released by stressed or dying cells, and an MI causes copious amounts of cell death and cytokine signalling, this proposes the idea that HMGB-1 is the IC ligand more representable of acute cardiac damage than other ligands.

To investigate potential involvement with the immune cells, we assessed TIM-3 expression. The adaptive immune system, i.e. CD8^+^ T cells and NK cells, downregulated TIM-3 gene expression already 24 hour post-MI. As TIM-3 is mostly known for its inhibitory effect in these cells, this would suggest that the cells lost their ability to be inhibited through this pathway over time^9^. Myeloid cells instead maintained TIM-3 expression throughout what is commonly accepted as the most inflammatory phase of an MI and only showed loss of TIM-3 expression after 8 weeks, during which fibrosis and remodeling is occurring^23,24^. In previous studies, TIM-3 expression by monocytes was inversely correlated with monocyte activation, wherein less TIM-3 means more monocyte activation, possibly to reduce the inhibition and allow clearance of injury/infection^25,26^. Together, this implicates the downregulation of TIM-3 as a pro-inflammatory process in the peripheral blood of patients after an MI.

snRNAseq data of cardiac tissue revealed an interesting connection between HMGB-1 on cardiomyocytes and TIM-3 in macrophages. Cardiac cells, including cardiomyocytes, significantly upregulated HMGB-1 gene expression, which we also observed on protein level in murine hearts having suffered ischemic injury. In human hearts, translocation of HMGB-1 suggests that even after the acute phase, HMGB-1 is still actively being shuttled throughout the cell and thus available for interaction. The ischemic zone showed most significant upregulation, which corroborates as this zone contains the most cells undergoing stress or cell death^27^. Interestingly, the myeloid cells were the only TIM-3 expressing cells predicted to interact with HMGB-1 from cardiomyocytes in all zones, most predominantly in the ischemic zone, as also shown by the spatial colocalization. While the expression of TIM-3 by non-T-cell immune cells is only recently appreciated, TIM-3 signalling of macrophages has been found to be of pro-inflammatory purpose, which is why we further subclustered the myeloid population. We found that of the three main types of macrophages – resident, pro-inflammatory, and anti-inflammatory – the pro-inflammatory *SPP1+* were not only the majority in the ischemic zone but also expressed the highest amount of TIM-3. This was validated by our in vitro data which demonstrated increased *SPP1* expression by M1 macrophages as opposed to unstimulated or M2 macrophages. The nature of the interaction between TIM-3 and HMGB-1 can be multifaceted. First, biologically, TIM-3 has been found to suppress polarization of macrophages from anti-inflammatory to pro-inflammatory through inhibition of NF-κB signalling^28,29^. What the effects of a ligand such as HMGB-1 binding to TIM-3 are on this balance is unknown. Presumably, binding of TIM-3 could alter signalling and allow for the macrophages to become pro-inflammatory. Previously, tumor cell TIM-3 interaction with Gal-9 in gliomas has been shown to be pro-inflammatory and modulation of this interaction has been proposed as a therapeutic target^30^. Our in vitro work also supports this theory, as stimulation with HMGB-1 induced M1 polarization by macrophages, which was prevented by blockade of TIM-3. Second, TIM-3 was found to be crucial in promoting phagocytosis in macrophages and DCs through interaction with PtdSer, facilitating clearance of apoptotic cells in the surroundings^31^. Binding of HMGB-1 here could inhibit this process, delaying resolution of inflammation^32^. Another possible mechanism circles around the result of HMGB-1 binding to its other receptors. Namely, it can also bind to TLRs which facilitate pro-fibrotic processes^33^. TIM-3 expression on innate immune system cells has in this setting been implicated as a possible decoy, or ‘’HMGB-1 sink”, to prevent activating TLRs pathways and subsequent pro-inflammatory responses^9^. We showed that HMGB-1-mediated M1-polarizating of macrophages likely does not occur through TLR4, and while RAGE blockade was able to prevent this in culture, the snRNAseq analyses only demonstrated interaction between HMGB-1 and TIM-3 in the ischemic zone, not between HMGB-1 and TLR4 or RAGE. Therefore, we believe that TIM-3/HMGB-1 binding in the heart occurs in the acute phase of an MI which can induce pro-inflammatory macrophages^30^.

Further mechanistic studies are needed to explore the interaction of cardiomyocyte-HMGB-1 with macrophage-TIM-3 and whether it poses as a therapeutic target.

## Methods

### Circulating levels of IC ligands

To determine the association of circulating levels of IC ligands with cardiac remodeling, plasma samples from patient enrolled in the GIPS-III trial, which has been previously described, were used^11,34^. In brief, the GIPS-III trial was designed to evaluate the effect of metformin on the preservation of left ventricular function in patients with MI but without diabetes. Inclusion criteria were being 18 years or older, an ST-elevation myocardial infarction (STEMI), and primary percutaneous coronary intervention (PCI) with implantation of at least one stent with a diameter of ≥3 mm resulting in TIMI flow grade 2 or 3 post-PCI. Major exclusion criteria were previous MI, diabetes, need for a coronary artery bypass graft surgery, severe renal dysfunction, and standard contraindications for magnetic resonance imaging (MRI).

Blood was collected upon admission and at 24 hours, 2 weeks, 7 weeks and 4 months follow-up. Cardiac MRI was conducted to determine infarct size and LVEF 4 months post-MI. LVEF was measured by MRI on a 3.0 T whole-body MRI scanner (Achieva; Philips) using a phased array cardiac receiver coil. An independent core laboratory (Image Analysis Center, VU University Medical Center, Amsterdam, the Netherlands) evaluated the MRI scans and assessed the primary efficacy measure, blinded for treatment allocation and clinical patient data.

Of the entire cohort of 379 patients, serum samples from 357 patients as well as standard laboratory assessment results were available. Circulating levels of HMGB-1 (NBP2-62766, Novus Biologicals), CEACAM1 (EHCEACAM1, Invitrogen) and Gal-9 (DGAL90, R&D Systems) were measured in serum samples according to manufacturers’ protocols. Associations of IC ligands serum levels with cardiac remodeling outcomes were assess with linear regression analyses. Measured concentrations were log-transformed before running the regression analysis. All variables with significant *p-*values (<0.05) in the univariable analysis were included in a multivariable regression analysis, using the backward elimination method which resulted in correction for multivariate regression adjusted for age, sex, BMI, hypercholesterolemia, TIMI flow and myocardial blush grade.

### scRNA sequencing of PBMCs

A post-hoc analysis was performed using scRNA sequencing data of PBMCs obtained from 38 patients with a first STEMI upon admission from the CardioLines study, and 38 sex- and age-matched healthy controls from the Lifelines study, as previously described^12,13,35^. In short, the MI cohort inclusion criteria were 18 years or older and presenting with a STEMI, as per European Society of Cardiology guidelines. PBMCs were isolated from whole blood samples collected upon hospitalization (hosp) and after 24 hours (24h) and 6–8 weeks (8w) and single cells were captured with 10x Chromium controller (10x Genomics) according to the manufacturer’s protocol. cDNA libraries were generated for v2 and v3 with the Single Cell 3’ Library & Gel Bead kit and i7 Multiplex kit according to the instructions. Subsequently, the libraries were sequenced on an Illumina NovaSeq6000 using a 150-bp paired-end kit. The sequencing data was demultiplexed, generated into FASTQ files, aligned to the hg19 reference genome, filtered based on cell and UMI barcodes, and count gene expressions per cell using CellRanger v3.0.2.

In Seurat (v.4.0) the output was processed seperately based on version chemistry (v2 or v3) and conditions (control or STEMI). SCTransform and PCA were run. To integrate the separate datapools, batch correction was performed using the Cannoncial Correlation Analysis (CCA) method within Seurat. The first 30 principal components were taken into consideration to identify shared neighbors taken as input for FindCluster and RunUMAP. To ensure quality of the data genes that were not detected in 3 cells or less were removed. Similarly, cells with a high percentage of mitochondrial genes, expressing <200 genes or >10 UMIs of the HBB gene were removed. Resulting in 129 873 cells for downstream processing^36^. The cells were annotated in each dataset separately using Seurat’s Azimuth method by projecting them on a previously annotated multimodal CITE-seq reference dataset of 162 000 PBMCs.

To visualize cell populations over time, cell population proportions were calculated by averaging cell population per patient and plotting relative expression in percentages. For the annotated dataset, aggregate expression in Seurat (v.5.0.0) was used to create pseudo-bulk profiles by summing up the count of cells belonging to the same donor bias^37,38^. DGE analysis was performed on the pseudo-bulk profiles, using the FindMarkers (test.use = DESeq2, logfc = 0.1, min.pct = 0.01) function^39^.

### snRNA sequencing and spatial transcriptomics of cardiac tissue

#### snRNA sequencing

To investigate IC expression and IC interactions in the infarcted myocardium, snRNAseq data was retrieved from an online available source (https://cellxgene.cziscience.com/collections/8191c283-0816-424b-9b61-c3e1d6258a77) as a Seurat v5 object^14^. Briefly, this study collected human myocardial tissue from non-transplanted donor hearts and patients with end-stage heart failure due to an MI, undergoing heart transplantation or implantation of a left-ventricular assist device. Tissues were sampled from the ischemic, border, remote and fibrotic zone. The latter was characterized as tissue from later staged after an MI from hearts exhibiting ischemic heart disease, representing a more long-term remodeling phenotype. Cardiac tissues were surgically sampled and immediately snap-frozen in liquid nitrogen, after which they were collected for single-nuclei isolation and sequencing using the Chromium Single cell 3’ reagent Kit v3 and Chromium i7 Multiplex Kit according to the manufacturer’s protocol. Cellranger (v6.0.2) was used to perform alignment, with the option ‘-include-intron’ to specify inclusion of intronic reads. In Seurat (v4.0.1) quality control, log normalization, dimensional reduction, principal component analysis (PCA) and harmony (v.1.0) integration was performed^36,40^. Clusters were determined by a Louvain algorithm and characterized by marker genes extracted from literature at the authors’ discretion. As an independent healthy reference, left ventricular snRNAseq data from https://www.heartcellatlas.org was used alongside the self-obtained control tissue^41^.

In total 191 795 annotated nuclei were retrieved. To determine transcriptomic differences between the sampling zones and healthy controls, Seurat’s (v.5.0.0) pseudo-bulking method was applied, accounting for donor and sampling zone, in combination with FindMarkers (test.use = DESeq2, logfc = 0.1, min.pct = 0.01)^39^. Cell population proportions for each zone were calculated by averaging cell population per patient and plotting relative expression in percentages. Ligand-receptor Analysis frAmework, LIANA, was used per sampling region and on control data to infer ligand-receptor communication^42^. LIANA enables the combination of multiple ligand-receptor analyses and recourses, providing a more robust prediction of ligand-receptor communication.

To further investigate myeloid subpopulations, these were isolated and reintegrated using harmony (v.1.2) within IntegrateLayers^40^. Findneighbors and RunUMAP were applied based on 30 dimensions. Clusters were defined on a resolution of 0.5 and characterized based on a combination of their original annotations, canonical marker genes and marker genes defined in the original study^14^. To compare macrophage subpopulations, differential gene expression was performed using a MAST statistical test, correcting for donor and sampling zone^43^. The significantly upregulated genes (*p* < 0.05) per myeloid subpopulation were used to perform a GO pathway analysis with clusterprofiler, correcting with FDR.

#### Spatial transcriptomics

To assess colocalization of predicted interactions provided by LIANA, we utilized spatial transcriptomic data from the same study, retrieved from https://explore.data.humancellatlas.org/projects/e9f36305-d857-44a3-93f0-df4e6007dc97^14^. In short, cardiac tissues had been sampled and frozen in liquid nitrogen, 10 μm tissue cryosections were stained with H&E and spatial gene expression slides were prepared according to the Visium User Guides (Visium, 10X Genomics). The sequencing data was pre-processed with SpaceRanger (v.1.3.2) and aligned to the hg38 reference genome. Low quality spots were filtered out and the count matrices were normalized with sctransform^44^. Cell2location was used to calculate the cell-type abundance per spot, with the hyperparameters: 8 cells per spot, 4 factors per spot, and 2 combinations per spot^45^. Spatial transcriptomic slides were retrieved as .rds files and analyzed in Seurat (v.5.0.0)^36^. Spots were marked as containing a cell type of interest if this cell type had an inferred abundance of 10% or higher. Subsequently, the normalized expression of HMGB-1 (*HMGB1*) and TIM-3 (*HAVCR2*) was assessed. If this was >1.5 and 1, respectively, the spots were labeled as such. Colocalization was then defined as the presence of both HMGB-1 and TIM-3 in the same deconvoluted spot.

### In vitro

#### Cell culture

THP1 monocytes (TIB-202, ATCC) were cultured in RPMI 1640 Medium with GlutaMAX™ Supplement (61870036, Life Technologies) with 10% Heat-Inactivated Fetal Bovine Serum (CA FBS-HI-11A) and 1% penicillin-streptomycin under sterile conditions at 37°C and 5% carbon dioxide (CO2). Differentiation of monocyte to macrophage was achieved by seeding 2 * 10^5^ THP1 monocytes in 12 wells-plates in medium with 150 nM PMA (P8139, Sigma-Aldrich)^46^. After 24 hours, the medium was replaced with RPMI without PMA and the macrophages were left to rest for another 24 hours.

Unstimulated macrophages, Mφ, were differentiated to polarize towards pro-inflammatory (M1) or anti-inflammatory (M2) macrophages (*n* = 6 for each group). For M1, cells were stimulated with 10 pg/mL LPS (L5293, Sigma-Aldrich) and 20 ng/mL IFN-γ (285-IF-100, R&D Systems). For M2, cells were stimulated with 20 ng/mL human recombinant IL-4 (130-093-917, Miltenyl Biotec) and 20 ng/mL human recombinant IL-13 (200-13, Thermofisher Scientific). After 48 h of stimulation, cells were harvested and analyzed. To determine whether treatment with HMGB-1 could induce a polarization towards M1 or M2, we treated Mφ macrophages with 5 µg/mL HMGB-1 (HM-101, HMGBiotech) for 48 h (*n* = 6 each).

For experiments in which TIM-3, TLR4 and RAGE were blocked before stimulation with HMGB-1, Mφ were incubated with either an antibody against TIM-3 (10 µg/mL AF2365, BioTechne), TLR4 (10 µg/mL, 14-9917-82, ThermoFisher Scientific) or RAGE (10 µg/mL, AF1145, BioTechne) for 1 hour before performing the experiment as indicated above (*n* = 3 each group).

#### Quantitative polymerase chain reaction (qPCR)

Cells were harvested in TRIzol (15596018, ThermoFisher Scientific) and RNA was extracted according to the manufacturer’s protocol. cDNA was created using the sensiFAST cDNA Synthesis Kit (BIO-65054, GC Biotech). qPCRs were performed using SYBR green for detection (primers listed in Supplementary Table 2). *Ct* values were used to calculate fold-change upon correction with housekeeping gene *RPS9*.

### Immunohistochemical analysis of HMGB-1

#### Murine in vivo study

MI experiments were performed in 6-week-old male mice with a C57Bl/6 J background (The Jackson Laboratory, Bar Harbor, ME, USA) as previously described^19^. Briefly, mice were intubated and mechanically ventilated under 2% isoflurane before undergoing surgery. MI was induced through permanent ligation of the LAD, whereas in sham-operated animals a suture was placed on the artery and removed without ligation. After a 6-week follow-up, mice were euthanized under 2% isoflurane anaesthesia by excising the heart, and the hearts were harvested in paraffin for immunohistochemical analyses. In total, immunohistochemistry analyses were performed on 3 MI and 3 sham-operated mice.

#### Human samples

Cardiac tissue was sampled from the left ventricle. Control samples (*n* = 3) were obtained from patients post-mortem, without cardiac cause. MI samples (*n* = 2) were obtained from patients who had endured an MI and required a heart transplantation.

#### Immunohistochemical staining and analysis

Murine cardiac tissue and left ventricular human cardiac tissue from two patients with ischemic injury and two patients with healthy hearts was fixed in 10% formaldehyde for 24 h, routinely embedded in paraffin, and cut into 5 μM sections. HMGB-1 (ab79823, Abcam) and anti-rabbit IgG secondary antibody (#8889, Cell Signaling Technology) immunohistochemistry was performed according to manufacturer’s instructions and tissue was imaged using a Hamamatsu NanoZoomer. Images were analysed using the Fiji processing package 2.15.1 of ImageJ.

### Statistical analyses

Transcriptomics data was analysed using RStudio and Seurat v5, with the appropriate analyses described above. Normal distribution of data was tested using the Shapiro-Wilk test. All analyses for ELISA and immunohistochemistry data were conducted using Stata 14.2 and GraphPad Prism 9.1.0. Normally distributed variables are presented as mean ± SEM and compared between two groups with the two-sample *t*-test, with 2-tailed significance set at *p* < 0.05. For in vitro data, an Ordinary one-way ANOVA was performed with a Dunnett test to correct for multiple comparisons, and significance set as *p* < 0.05. All images were created using BioRender, GraphPad Prism or Adobe Illustrator.

### Study approval

The GIPS-III trial (NCT01217307), Lifelines and CardioLines were executed in accordance with the Declaration of Helsinki and were approved by the local ethics committee in Groningen and the national regulatory authorities as previously described^11–13,34,35^. snRNAseq data of human myocardial tissue was obtained from a publicly available dataset, of which in the original article is stated that everything was conducted in adherence to local and European regulatory authorities^14^. For the human cardiac tissue samples from patients with ischemic injury, informed consent was obtained from all patients for being included in the study according to the protocol approved by the Local Ethics Committee (IK-NP-0021-24/1426/14) in Warsaw, Poland. Written informed consent was obtained from all participating subjects and patients.

All murine experimental procedures were performed in accordance with the local and European Union guidelines (DEC 6844, the Netherlands) and (BMWF-66011_0063-II_10b_2010, Austria).

## Supplementary information

Supplementary Data

Supplementary figures and tables

## Data Availability

The single cells RNA sequencing data on PBMCs is available at https://ega-archive.org/datasets/EGAD00001010064. The cardiac tissue data analysed during this study are obtained from https://cellxgene.cziscience.com/collections/8191c283-0816-424b-9b61-c3e1d6258a77 and https://explore.data.humancellatlas.org/projects/e9f36305-d857-44a3-93f0-df4e6007dc97.
