# Supplementary material for: The immune checkpoint TIM-3/HMGB-1 axis in myocardial infarction
Source: NPJ Cardiovasc Health. 2025 Jun 27;2:32. doi: 10.1038/s44325-025-00061-x (PMC12446057; doi:10.1038/s44325-025-00061-x)

**Supplementary figures and tables**

**Supplementary table 1** Associations of CTLA-4 and PD-1 ligands with cardiac remodelling 4 months post-MI.

|  | **Infarct size** | | | | | | **LVEF** | | | | | | |
| --- | --- | --- | --- | --- | --- | --- | --- | --- | --- | --- | --- | --- | --- |
|  | **Univariate** | | | **Multivariate** | | | **Univariate** | | | **Multivariate** | | | |
|  | ***β*** | ***p-value*** | | ***β*** | ***p-value*** | | ***β*** | ***p-value*** | | ***β*** | ***p-value*** | | |
| **CTLA-4** | | | | | | | | | | | | | |
| **CD80** | -0.179 | | 0.059 | - | | - | 0.137 | | 0.150 | - | | - |  |
| **CD86** | -0.176 | | 0.062 | - | | - | 0.521 | | 0.574 | - | | - |  |
| **PD-1** | | | | | | | | | | | | |  |
| **PD-L1** | 0.039 | | 0.680 | - | | - | -0.066 | | 0.439 | - | | - |  |
| **PD-L2** | -0.046 | | 0.617 | - | | - | 0.026 | | 0.798 | - | | - |  |

**Supplementary table 2** Primers sequences for qPCR.

| IL-6 forward | AAGCCAGAGCTGTGCAGATGA |
| --- | --- |
| IL-6 reverse | GCTGGCATTTGTGGTTGGGT |
| CXCL10 forward | GGTGAGAAGAGATGTCTGAATCC |
| CXCL10 reverse | GTCCATCCTTGGAAGCACTGCA |
| CCL18 forward | CATGGCCCTCTGCTCCTGTG |
| CCL18 reverse | CCGGCCTCTCTTGGTTAGGAG |
| RPS9 forward | TTGCTTAGGCGCAGACGGG |
| RPS9 reverse | GACCCTCCAGACCTCACGTT |
| FN forward | GGACCAGGACCAACAAAAAC |
| FN reverse | AGACACTAACCACATACTCCAC |
| SPP1 forward | GGCCAGTTGCAGCCTTCTCA |
| SPP1 reverse | GTGGCCACAGCATCTGGGTA |
| HAVCR2 forward | GTTGGTCATCAAACCAGCCAAGG |
| HAVCR2 reverse | ATCCTTGGAAAGGCTGCAGTG |
| TLR4 forward | GTTGGTCATCAAACCAGCCAAGG |
| TLR4 reverse | ATCCTTGGAAAGGCTGCAGTG |
| AGER forward | CTGGGTGCTGGTCCTCAGTC |
| AGER reverse | TGGGGAGACAGGACCTTCCA |
| CASP1 forward | GCTGAGGTTGACATCACAGGCA |
| CASP1 reverse | TGCTGTCAGAGGTCTTGTGCTC |
| IL1B forward | GAAGTACCTGAGCTCGCCAGT |
| IL1B reverse | CTGCCTGAAGCCCTTGCTGT |
| IL18 forward | CCAGATCGCTTCCTCTCGCA |
| IL18 reverse | TTCCAGGTTTTCATCATCTTCAGC |

**Supplementary figure 1**Marker genes for validation of PBMC cell type clusters

**
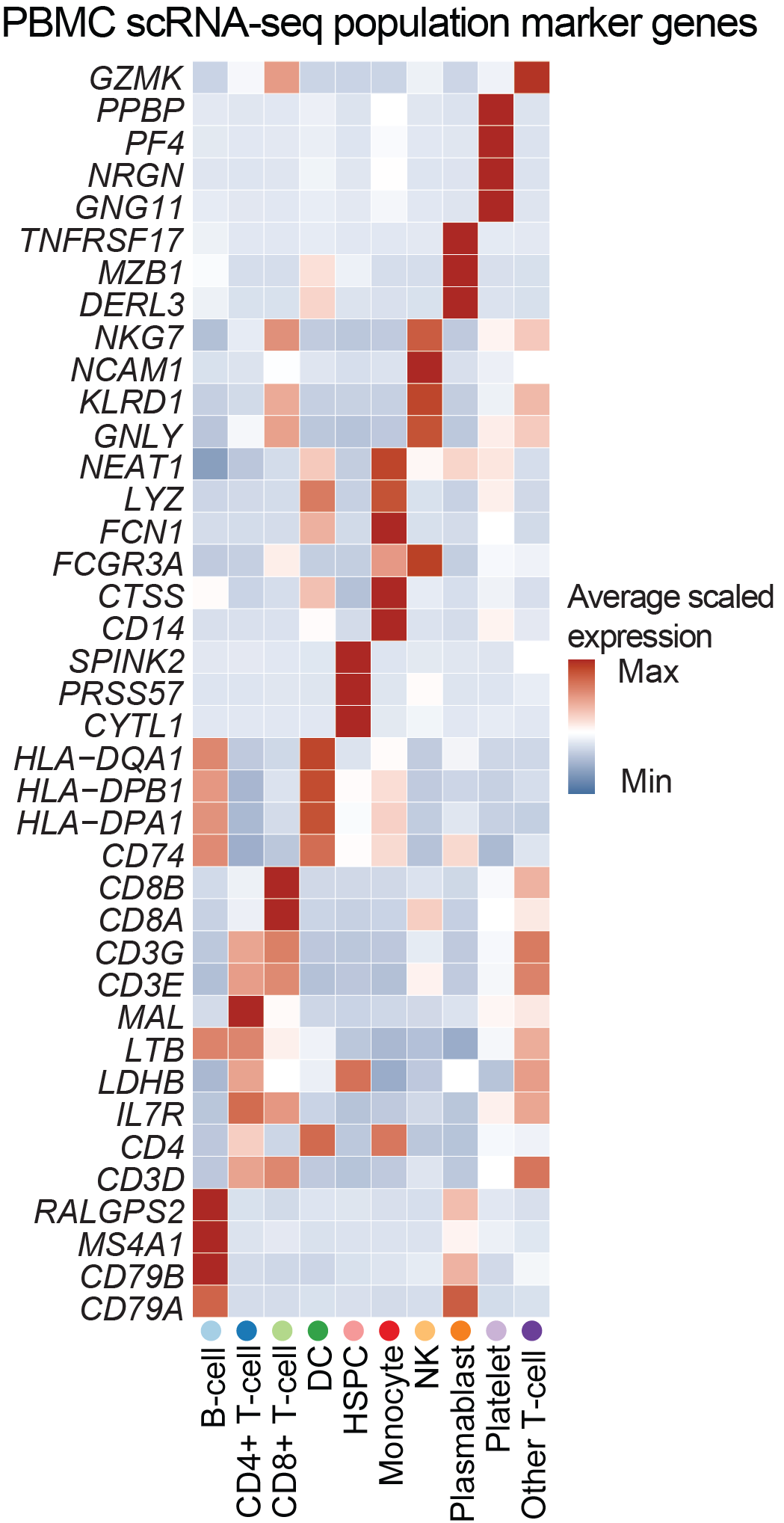
**


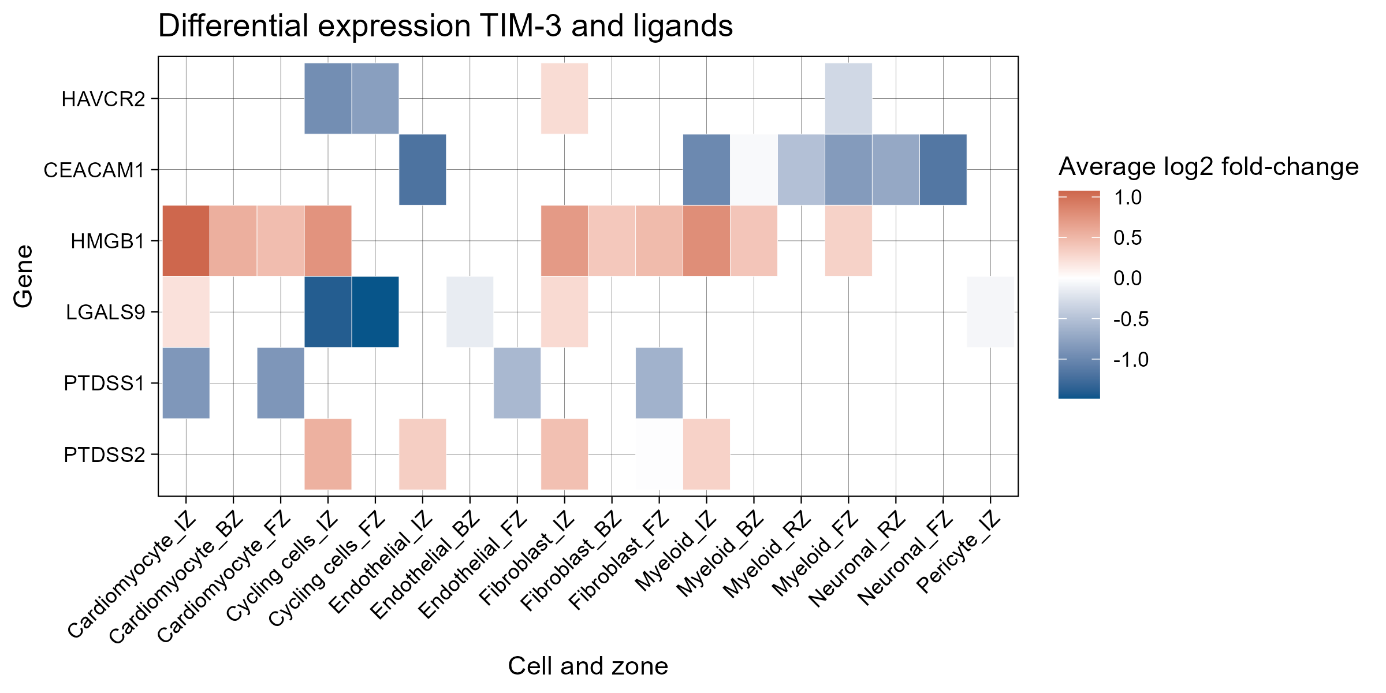
 **Supplementary figure 2** Heatmap of differentially expressed TIM-3 and ligands in all cell populations in all zones

**Supplementary figure 3** Heatmap of marker genes for myeloid cell populations


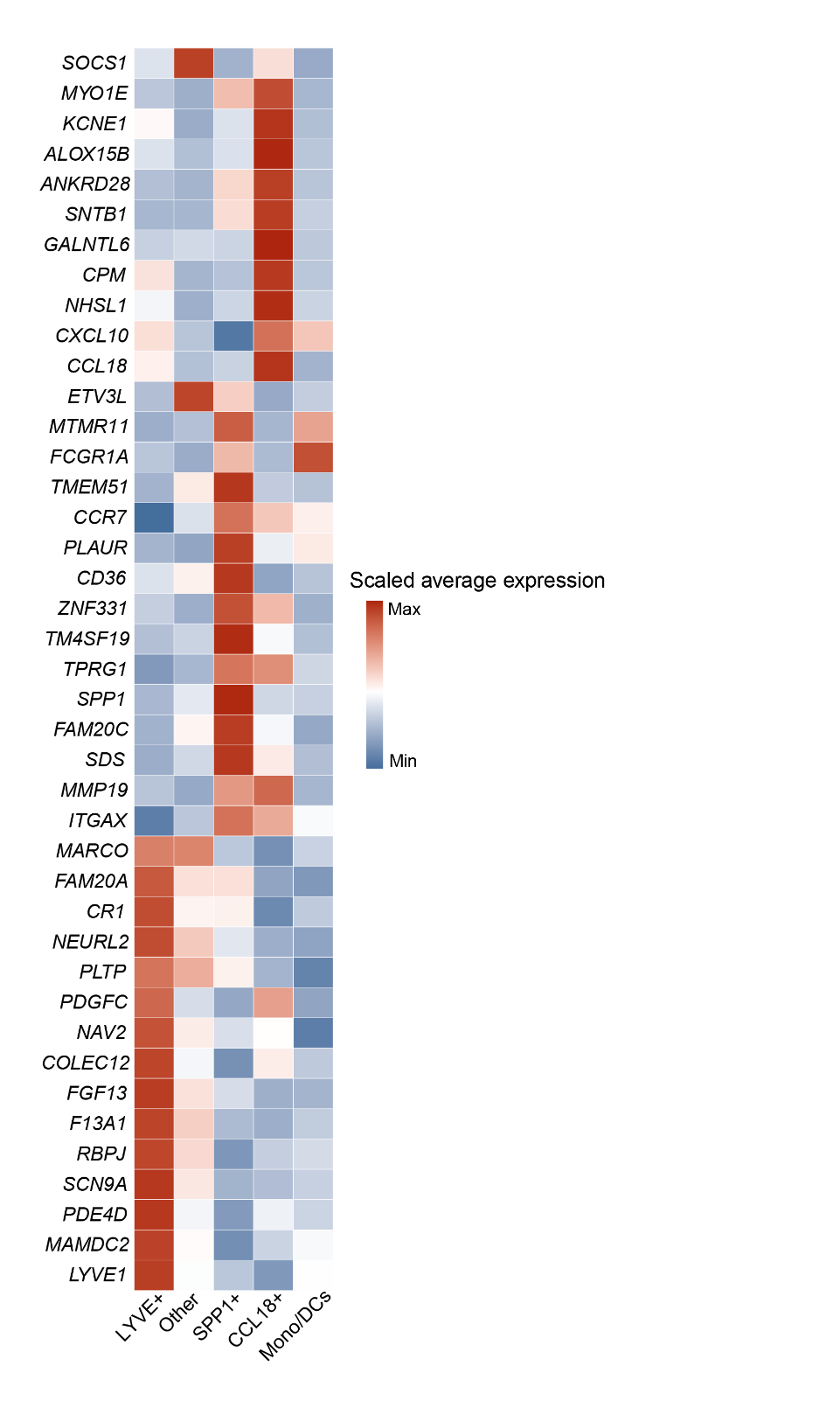

Supplement: Supplementary file 2 — Supplementary figures and tables [file 44325_2025_61_MOESM2_ESM.docx]
